# Poor readers' retrieval mechanism: efficient access is not dependent on reading skill

**DOI:** 10.3389/fpsyg.2015.01552

**Published:** 2015-10-16

**Authors:** Clinton L. Johns, Kazunaga Matsuki, Julie A. Van Dyke

**Affiliations:** ^1^Haskins LaboratoriesNew Haven, CT, USA; ^2^Department of Linguistics and Language, McMaster UniversityHamilton, ON, Canada

**Keywords:** memory retrieval, sentence processing, speed-accuracy trade-off, reading comprehension, individual differences

## Abstract

A substantial body of evidence points to a cue-based direct-access retrieval mechanism as a crucial component of skilled adult reading. We report two experiments aimed at examining whether poor readers are able to make use of the same retrieval mechanism. This is significant in light of findings that poor readers have difficulty retrieving linguistic information (e.g., Perfetti, 1985). Our experiments are based on a previous demonstration of direct-access retrieval in language processing, presented in McElree et al. (2003). Experiment 1 replicates the original result using an auditory implementation of the Speed-Accuracy Tradeoff (SAT) method. This finding represents a significant methodological advance, as it opens up the possibility of exploring retrieval speeds in non-reading populations. Experiment 2 provides evidence that poor readers do use a direct-access retrieval mechanism during listening comprehension, despite overall poorer accuracy and slower retrieval speeds relative to skilled readers. The findings are discussed with respect to hypotheses about the source of poor reading comprehension.

## Introduction

The ability to comprehend written language is an enormously important skill, as shown by robust correlations between poor reading comprehension and a variety of undesirable consequences, including constrained economic mobility, reduced economic success, and increased risk of poor health outcomes (Kutner et al., 2007; National Institute for Literacy, 2008). Many models of text processing (e.g., Kintsch, 1988, 1998; Myers and O'Brien, 1998; van den Broek et al., 1999; for reviews see Long et al., 2006; McNamara and Magliano, 2009), sentence processing (Gibson, 2000; Van Dyke and Lewis, 2003; for review see Van Gompel, 2013), and reading disability (e.g., Hogaboam and Perfetti, 1975; Shankweiler and Crain, 1986) incorporate the idea that comprehension is constrained by the architecture of the human memory system. Given this, it is important to understand the interaction between memory mechanisms—such as retrieval—and the sentence parsing processes on which successful comprehension depends. Previous research has shown that university students employ a content-addressable, direct-access mechanism to efficiently retrieve information from memory during reading (e.g., McElree and Dosher, 1989; McElree, 2001; McElree et al., 2003; for reviews see McElree and Dyer, 2013; McElree, 2015). In this article, we assess the potential relation between reading skill and memory retrieval. We report two speed-accuracy tradeoff (SAT) experiments that (1) validate an auditory implementation of the technique for the assessment of the dynamics of memory retrieval, and (2) investigate whether poor readers, like skilled readers, are able to employ a content-addressable direct-access retrieval mechanism during online auditory sentence comprehension. Our goal is to determine whether poor comprehenders possess the architectural primitives that are known to support skilled reading comprehension.

Models of sentence parsing typically offer richly detailed accounts of the linguistic processes that drive parsing operations. Examples of such operations include heuristic routines (e.g., minimal attachment, late closure, main assertion preference, active filler strategy); serial (or parallel) control structures, which may or may not activate (or inhibit) competing interpretations; ranked vs. unranked consideration of extra-syntactic (e.g., semantic, referential, pragmatic, visual) information; and so on. When these processes go awry, errors are often either explicitly or implicitly associated with increased demands on the memory system, which are assumed to yield suboptimal application of these parameters. To illustrate, consider sentences (1) and (2), from a study by Frazier and Rayner (1982):

(1) While Susan was dressing the baby played on the floor.
(2) Since Jay always jogs a mile seems like a short distance to him.

Sentences such as (1) and (2) are thought to tax the memory system because an accurate parse, and consequent comprehension, is only possible by violating initial syntactic commitments (licensed by minimal attachment in (1) and late closure in (2); Frazier, 1979, 1987; for review, see Frazier, 2013). In such cases the parser must construct, assess, abandon, and reconstruct entire syntactic structures, and reassessment is assumed to involve costly search and diagnosis processes (e.g., Fodor and Inoue, 1994, 1998, 2000). Or, alternatively, it could be the case that the parser constructs and actively maintains multiple syntactic structures during online processing, the extra burden of which leads to processing difficulty (e.g., MacDonald et al., 1994). Furthermore, it is not just parsing errors that tax the memory system during language processing; complexity effects, in which more complex syntactic structures are claimed to be more memory-intensive, have been widely studied. A classic example is the difference in processing elicited by unambiguous sentences such as (3), which contains an object-extracted relative clause, and (4), in which the embedded subject-extracted relative clause results in a simpler syntactic structure (from King and Just, 1991):

(3) The banker that the barber praised climbed the mountain.
(4) The banker that praised the barber climbed the mountain.

In (3), it is thought that the initial filler noun phrase (*the banker*) must be actively maintained during the processing of the embedded clause, after which it may be integrated with the matrix verb *climbed* (e.g., via an active filler strategy; Clifton and Frazier, 1989); in contrast, (4) elicits no such active maintenance, and consequently is less demanding of the memory system.

These examples highlight the fact that the centrality of memory operations during parsing is both widely acknowledged and uncontroversial. In spite of this, theories of parsing (and text processing) are frequently vague regarding the memory mechanisms that support their finely-specified linguistic operations. Further, when a memory component has been elaborated, the focus has been almost entirely on the *storage* component, which is conceptualized as a limited-capacity working memory (WM) system (e.g., Just and Carpenter, 1992; Caplan and Waters, 1999; see also Daneman and Carpenter, 1980). In this system, the dynamic allocation of resources between language and memory operations must support incremental parsing operations, maintenance of critical sentential information, and retrieval of information from both WM and long-term memory (LTM). A fundamental tenet of this approach is that information required for interpretation must be maintained in an active, highly accessible state, and when this is difficult—perhaps owing to low capacity, or to increased computational costs, or both—processing suffers. There is no shortage of research whose findings have been interpreted as evidence for a capacity-based memory architecture (e.g., King and Just, 1991; Fedorenko et al., 2006; Nieuwland and Van Berkum, 2006, among many others). Implicit in these approaches is the idea that stored information is accessed via a serial search process (Just and Carpenter, 1992; Gibson, 1998, 2000): thus, the greater the amount of linguistic material intervening between dependent constituents (which must, therefore, be searched), the more difficult a given construction will be.

Despite the abundance of psycholinguistic studies that adopt this conception of memory, there is substantial disagreement about the nature of the unit of “active maintenance” that defines these search processes. Various proposals have characterized it as words (Warner and Glass, 1987), discourse referents (Gibson, 2000), incomplete grammatical dependencies (Abney and Johnson, 1991; Gibson, 1998), syntactic embeddings (Miller and Chomsky, 1963), or representations of entire alternative syntactic structures (Just and Carpenter, 1992; MacDonald et al., 1992). The fact that consensus has been elusive indicates the weakness of this approach. In addition, significant practical concerns exist, such as poor test-retest reliability of metrics designed to gauge WM capacity (Waters and Caplan, 2003), and collinearity with many other cognitive measures (e.g., Van Dyke et al., 2014). Further, the approach has also been questioned on theoretical grounds; for example, innate capacity differences that limit comprehension ability could emerge naturally from individual linguistic experience rather than from a separable memory system (e.g., MacDonald and Christiansen, 2002).

However, the most fundamental objection to assuming that a search-based limited-capacity memory mechanism supports language comprehension derives not from the need to reconcile these kinds of inconsistencies, but from the disparity between the proposed WM architecture and the empirical evidence regarding the memory structures and operations themselves. For example, there is substantial evidence that the amount of information that can be maintained in an active, accessible state is far more constrained than has been assumed by any parsing architecture supported by a fixed-capacity WM system. Memory studies using the SAT method report that only a single item (i.e., the last item processed) is actively maintained, meaning that only this item would not require retrieval (McElree, 1998, 2001, 2006; McElree and Dosher, 2001)^1^. All other items—that is, items that should be both *within* as well as *outside of* a traditional WM span—are accessed 30–50% more slowly than the active item (Wickelgren et al., 1980; McElree, 1996, 1998). Results such as these clearly indicate that the capacity of active memory is limited to information that is currently in the focus of attention, while information that is outside focal attention is passively represented. Moreover, items that are outside of focal attention are accessed with constant speed, regardless of how recently they occurred in relation to the retrieval probe. This pattern is consistent with the operation of a cue-based, direct-access retrieval mechanism in which all available cues are matched simultaneously, with the degree of featural overlap between the target and the available retrieval cues determining retrieval success (for a review see Clark and Gronlund, 1996). While language processing with such a severely constrained active memory capacity may seem implausible, the feasibility of a processing architecture in which only the most recent item remains in focal attention has been demonstrated in an implemented computational model (Lewis and Vasishth, 2005; Lewis et al., 2006). Within this architecture, it is the direct-access mechanism that provides the computational power to compensate for the severely constrained memory capacity. Indeed, there are now a number of studies, across a broad range of sentence constructions, that provide evidence for direct access in language processing (e.g., McElree, 2000; McElree et al., 2003; Martin and McElree, 2008, 2009, 2011; Van Dyke and McElree, 2011; for reviews see McElree, 2006, 2015).

The paradigmatic evidence for direct-access retrieval in sentence processing was provided by McElree et al. (2003), who asked university students to read sentences containing grammatical dependencies in which the distance between the grammatical head (e.g., *book*) and its dependent (e.g., *ripped*) was manipulated:

(5) The *book ripped*.
(6) The *book* that the editor admired *ripped*.
(7) The *book* that the editor who quit the journal admired *ripped*.

McElree and colleagues found that as the amount of material interpolated between the matrix verb and the sentential subject increased, the probability of accurate retrieval decreased: participants responded very accurately in (5), less accurately in (6), and still less accurately in (7). If “book” were accessed via a serial search mechanism, similar systematic differences should also have been observed in indices of retrieval speed; that is, a serial search mechanism also predicts that participants should be fastest to access *book* in (5), slower to access *book* in (6), and slower still in (7). Instead, McElree and colleagues found that participants resolved the *book*-*ripped* dependency very quickly in sentences such as (5), and with a slower—but constant—speed in (6) and (7)^2^. Thus, although the memory representations did vary in their availability (perhaps because of decay, or reduced distinctiveness as the number of NPs increased, or both), participants used the cues provided by the verb (e.g., selectional information) to guide direct retrieval of the appropriate NP from memory. Crucially, these results are not compatible with a serial search-based retrieval mechanism, which predicts that items that vary in their availability should not be accessed with equal speed. Hence, this study clearly shows that the collegiate readers were not engaging in a serial, backwards search through information that is no longer active in memory.

In light of this evidence, it seems plausible to suggest that content-addressable, direct-access retrieval is a fundamental property of the human language faculty, and that a cue-based retrieval parser (e.g., Van Dyke and Lewis, 2003; see also Lewis and Vasishth, 2005; Lewis et al., 2006) is the “default” processor for linguistic input. However, there are two potential objections to this proposal. First, all of the studies attesting to this type of retrieval during language processing have employed visually presented stimuli. That is, these studies only provide evidence that a cue-based retrieval parser is active during *reading*; it remains possible that processing *spoken* language initiates qualitatively different memory operations than those observed in reading tasks. The presence of orthographic information could enhance encoding and access during reading in ways that would necessarily be absent during listening comprehension (e.g., Harm and Seidenberg, 2004)—a potential confound that is amplified by extensive evidence that deficient orthographic decoding plays a role in reading difficulty (Shankweiler and Crain, 1986; Bell and Perfetti, 1994; Long et al., 2006). Second, these studies have uniformly tapped university subject pools for their participants, with the result that evidence for the cue-based retrieval parser comes entirely from relatively skilled readers. This raises the possibility that cue-based, direct-access retrieval develops concomitantly with reading skill; that is, more reading or language experience may “tune” the parser to make it more efficient, while less skilled readers may employ less efficient (e.g., search-based) memory operations during language comprehension. Such an account is consistent with some models of WM that suggest that efficient retrieval is predicated on efficient access structures that are derived from acquisition of skill proficiency (e.g., Ericsson and Kintsch, 1995).

## Experiment 1

Our first experiment examines memory retrieval during auditory language comprehension. This is crucial for assessing whether both auditory and written language processing use direct-access retrieval, as well as for studying retrieval mechanisms in poor readers, whose poor orthography-to-phonology decoding represents an important confound for any study implemented in the visual modality. We created an auditory implementation of the SAT procedure, in which participants listened to, and responded to, a series of sentences that either were or were not grammatically acceptable.

The SAT procedure provides an unambiguous estimate of access speed, which is required to differentiate direct-access retrieval from serial search processes. This contrasts with more commonly used timing measures, such as reaction and reading times, which are not “process pure”: in these paradigms, slower RTs may occur as a result of either actual speed differences, differences in the relative likelihood that information will be successfully recovered from memory, or both. In addition, these measures are vulnerable to idiosyncratic response criteria—that is, participants can adopt liberal or conservative response patterns, emphasizing accuracy at the expense of speed, or speed at the expense of accuracy (see McKoon and Ratcliff, 1992; McElree, 1993; Ratcliff and McKoon, 2008). In contrast, the SAT procedure permits the assessment of both by computing response functions that model the entire time course of information accrual (Wickelgren, 1977). The SAT procedure's fine-grained assessment of retrieval dynamics forms the basis for all unambiguous evidence that a fast, content-addressable, direct-access retrieval mechanism with a single-item focal span supports typical online language comprehension processes (e.g., McElree and Griffith, 1995, 1998; McElree, 2000; McElree et al., 2003; Foraker and McElree, 2007; Martin and McElree, 2008, 2009, 2011; Van Dyke and McElree, 2011; for reviews see Foraker and McElree, 2011; McElree, 2015).

Our goal in Experiment 1 was to validate our auditory implementation of the SAT technique by replicating Experiment 2 of McElree et al. (2003) with a comparable population (university students) using auditory versions of the stimuli from that study. Consistent with that study, we predicted that access would be fastest when the critical item was still active in the focus of attention (i.e., the most recently processed word). If the speed of access in the longer conditions, in which retrieval is necessary, is invariant, this supports an account of listening comprehension in which direct-access retrieval is used. However, if retrieval speed in the longer conditions varies systematically according to the distance between the retrieval cue and its target, this would support a search-based retrieval mechanism.

### Method

#### Participants

Informed consent was obtained from five undergraduates at Yale University. The participants were right-handed native English speakers, and were paid for their participation ($20/h). Each participated in one 1-h SAT training session, followed by two 3-h experimental sessions; these sessions were comprised of two 1-h SAT sessions (for a total of four), separated by a 1-h period in which they completed additional cognitive assessments (for a separate study) and rested. Details about the training and experimental sessions are described below.

#### Materials

Materials were adapted from those used in Experiment 2 of McElree et al. (2003). These constructions permit assessment of the speed and accuracy with which a matrix intransitive verb (e.g., *ripped, laughed*) retrieves its grammatical subject noun (e.g., *book*); examples appear in Table 1. Because we planned to test a population with a wide range of comprehension ability in our second experiment, our materials did not include all of the conditions presented in McElree et al. We selected a subset of conditions that linearly increased the surface distance, and the corresponding time, between each sentence's subject NP and matrix verb. For each item, participants were required to determine whether the subject-verb relation was either acceptable or unacceptable (see Procedure and Data Analysis, below). The conditions in both this and the next experiment are:

**Table 1:** **Constructions used in Experiments 1 and 2 (adapted from McElree et al., 2003)**.

**Construction**	**Acceptability**	**Example**
No Interpolation	Acceptable	T1. The book ripped.
No Interpolation	Unacceptable	T2. The book laughed.
Object relative	Acceptable	T3. The book that the editor admired ripped.
Object relative	Unacceptable	T4. The book that the editor admired laughed.
Object relative	Unacceptable	T5. The book that the editor amused ripped.
Object relative + subject relative	Acceptable	T6. The book that the editor who quit the journal admired ripped.
Object relative + subject relative	Unacceptable	T7. The book that the editor who quit the journal admired laughed.
Object relative + subject relative	Unacceptable	T8. The book that the editor who quit the journal amused ripped.

*No Interpolation* (T1 and T2): in the shortest conditions, the subject and verb are directly adjacent to each other (no retrieval needed).

*Interpolated Object Relative Clause* (T3 and T4): distance between subject NP and verb is increased by four words. T3 and T4 are identical to T1 and T2 with the exception of the additional embedded clause.

*Interpolated Object and Subject Relative Clauses* (T6 and T7): in the longest conditions, the subject and verb are separated by eight words. T6 and T7 are identical to T3 and T4 with the exception of the additional embedded subject relative clause.

*Additional processing encouragement* (T5 and T8): as in McElree et al. (2003), we included a second type of unacceptable item in each of the longer conditions. These items, exemplified by T5 and T8, are identical to the other items in their corresponding conditions with one exception. In these items, the grammatical inconsistency that determined acceptability was located in the interpolated information. For example, as shown in Table 1, although the embedded transitive verb requires a direct object, *book* is not an acceptable argument for *amused*. These types of sentences were included to encourage our participants to attend to (and process) the interpolated material.

We selected 48 instances of each of the eight types of sentence (T1–T8, i.e., three acceptable and five unacceptable) from the original materials used in McElree et al. (2003). This yielded a total of 384 experimental items, which we edited slightly in order to make the vocabulary level more appropriate to the participants in our second experiment. From this set of items, we generated four experimental lists of 96 sentences. Each list was comprised of 12 instances of each sentence type. Participants listened to one list during each of the four SAT sessions.

#### Procedure

All stimuli were randomized within each testing session and presented using the E-Prime experimental package (Schneider et al., 2002). Unlike the original study in which a single-response Speed Accuracy Tradeoff (SR-SAT) paradigm was used, we adopted the multiple-response Speed-Accuracy Tradeoff (MR-SAT) method (Wickelgren et al., 1980; McElree, 1993; see also Bornkessel et al., 2004; Foraker and McElree, 2007; Van Dyke and McElree, 2011). Because more responses are collected per trial, MR-SAT paradigms require fewer items, and consequently fewer experimental sessions to complete an experiment. Each trial began with the words “Listen carefully,” which appeared in the center of the screen throughout the trial. The initial appearance of these words was accompanied by an auditory fixation cue (a tone). This cue alerted participants to the imminent auditory presentation of a sentence, which began 500 ms after the offset of the cue. All sentences were prepared using version 2.0.3 of Audacity® recording and editing software (Audacity Team, 2015; http://audacity.sourceforge.net). The sentences were presented at a natural speaking rate (in contrast to previous visual SAT studies, in which sentences were segmented word-by-word or phrase-by-phrase). A sequence of 15 tones (100 ms, 1000 Hz, every 350 ms) was spliced into the sentence recording, beginning 200 ms prior to the onset of the sentence-final critical word. The tones were presented simultaneously with and following the critical word, forming a 5000 ms response period. Participants were instructed to judge whether each sentence was an acceptable English sentence. They were trained to press the response key(s) corresponding to their acceptability judgment in time with the tone sequence. At the onset of the tones, participants began responding by pressing both response buttons, indicating that they did not yet know whether or not the sentence was acceptable. After hearing the sentence-final word, participants indicated whether the sentence was acceptable or unacceptable by choosing either the YES or NO response key, and continuing to press only that button in time with the tones.

During the training session, participants first heard and responded to response tones in isolation, in order to become familiar with the auditory and motor aspects of the SAT procedure; they subsequently heard and responded to practice items similar to those in the experiment. In addition to the initial training, participants also completed a 15-min refresher session at the beginning of the second experimental session in order to refamiliarize themselves with the task. Participants received feedback about their responses in both training sessions, indicating whether their responses were faster or slower than, or out of sync with, the rhythm of the response tones. In addition, they were taught that they could change their response; for example, if at first they decided that a sentence was acceptable (and consequently stopped pressing the NO response key while continuing to press the YES response key), but subsequently changed their mind and deemed it unacceptable, they could switch their response (i.e., stop pressing YES, and resume pressing NO). Participants were taught that they could change their response at any time—and multiple times, if necessary—during the 5000 ms response period.

#### Data analysis

SAT data provide indices of both accuracy and speed associated with responses. In studies using the SAT method, a stable SAT function can be calculated for each participant and, as a consequence, each participant is analyzed separately. This approach has two advantages: it reduces the variance associated with each participant's data, and it minimizes distortion associated with averaging across participants. Consistent patterns that emerge across participants are subsequently considered through analyzing both modeling consistency across individuals and modeling of the averaged data.

Accuracy was computed for each time point in the response period using a standard measure of sensitivity (*d*′). Potential response bias was controlled by calculating *d*′ using z-scores for hits and false alarms [*d*′ = *z*(hits)−*z*(false alarms)]. In this experiment, a “hit” is a YES response to an acceptable sentence, and a “false alarm” is a YES response to an unacceptable sentence.

The asymptote, rate, and intercept for each response function were assessed by fitting the *d*′ accuracy scores at each response point (*t*), with an exponential approach to a limit:

$$\begin{array}{l} d\prime (t)=\lambda (1-e^{-\text{β}(t-\delta )})\;\text{for}\;t>\delta ,\;\text{ else}\;\;0 \end{array}$$

Thus, *d*′ is the result of the interaction of the two factors that define an SAT function: the asymptote of the function (λ), and the speed with which that asymptote is reached. Speed is jointly determined by two distinct parameters: the intercept of the function (δ), which is the point at which response accuracy rises above chance, and the rate at which response accuracy reaches asymptotic performance (β). Calculated *d*′ scores are then fit to hierarchically nested models ranging from a null model, in which the experimental conditions are fit using a single asymptote, rate, and intercept, to a fully saturated model, in which the conditions are each fit with a unique set of parameters. For data modeling, we used functions from the package *mrsat* (Matsuki et al., in preparation)^3^. The fitting function applied four different optimization algorithms that are implemented in R functions: (1) an iterative hill-climbing algorithm (Reed, 1976) similar to STEPIT (Chandler, 1969), which has been used in the majority of previous SAT studies of language processing and is implemented in the *acp* function; (2) a limited-memory Broyden–Fletcher–Goldfarb–Shanno algorithm with box constraints (Byrd et al., 1995) implemented as a part of the *optim* function; (3) a box-constrained optimization algorithm based on PORT routines developed by Bell Labs (Fox et al., 1978) as implemented in the *nlminb* function; (4) an unconstrained optimization algorithm based on a Newton-type method implemented in the *nlm* function (Dennis and Schnabel, 1983; Schnabel et al., 1985). Each of these algorithms were applied 10 times with randomly chosen starting parameter values on each run, and the resulting set of parameters that provided the best model fits were selected. Fit quality was assessed in two ways. First, we calculated a modified *R*^2^ statistic, in which the number of parameters present in each model is used to adjust the proportion of variance accounted for by each model (Judd and McClelland, 1989). Second, we evaluated the consistency of the parameter estimates across participants.

All SAT response function and statistical analyses were carried out with the R statistical software, version 3.2.1 (R Core Team, 2015). For analyses, we used the package *lme4* (Bates et al., 2015). We used linear mixed-effect regression (*LMER*; Baayen, 2004, 2008; Baayen et al., 2008) to assess the observed empirical data and the fitted parameter estimates for each of the candidate models described in the Results Section. Mixed-effects models included fixed effects of Construction and random intercepts for participants. For evaluation of the main effect of Construction, we report the associated *F*-value, as well as the denominator degrees of freedom and *p*-values that were calculated based on Satterthwaite's approximation using the *lmerTest* package (Kuznetsova et al., 2015). We also report the *t-*values associated with our analyses, adopting the convention whereby any effect whose absolute *t-*value exceeds 2 is considered significant (Gelman and Hill, 2007).

### Results

**Figure 1:**
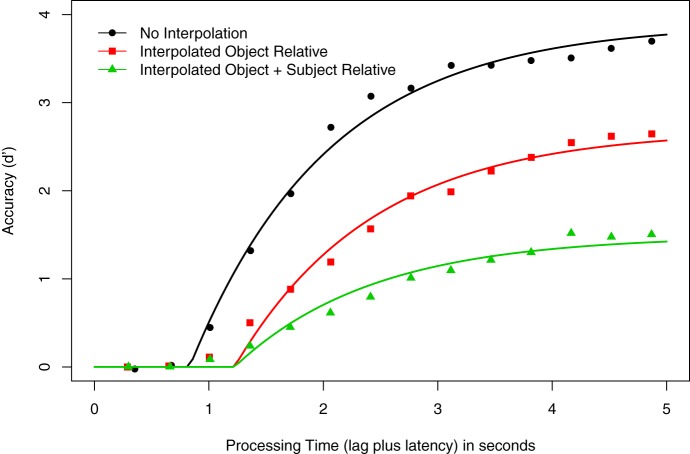
**Speed-accuracy tradeoff results for Experiment 1**. Average *d*′ accuracy as a function of processing time (in seconds) for the grammaticality judgments of sentences with the following constructions: no embedded material (circles), one object-relative clause (squares), and one object- and one subject-relative clause (triangles). Smooth curves show the 3λ-1β-2δ exponential model (see text of Experiment 1).

Figure 1 shows the averaged *d*′ data at each response point, as well as smoothed curves depicting the best fitting model (3λ-1β-2δ; see below) as a function of processing time for the three Construction conditions (No Interpolated Material, Interpolated Object Relative, Interpolated Object + Subject Relative). As in McElree et al. (2003), visual inspection of the data suggests that asymptotic accuracy is negatively correlated with the amount of material interpolated between the matrix verb and its subject. This observation is supported by the LMER analysis of the mean of the last four *d*′ values, which is the empirical estimate of asymptotic accuracy. This confirmed a main effect of Construction, *F*_(2, 8)_ = 40.17, *p* < 0.001. Pairwise comparisons showed that accuracy was higher when there was no material between subject and verb (*d*′ = 3.58) than when there was an intervening object relative clause (*d*′ = 2.55), *t* = −4.33, or when there were intervening subject and object relative clauses (*d*′ = 1.45), *t* = −8.96. In addition, the asymptotic accuracy of the Interpolated Object Relative condition was significantly higher than that of the Interpolated Object + Subject Relative condition, *t* = −4.63. This pattern of results replicates the pattern reported in McElree et al. (2003) for these conditions.

Initial hierarchical modeling of the data assessed three models, differing only by the number of asymptote parameters assigned to the models. First, we assessed the null model, in which a common asymptote (λ), rate (β), and intercept (δ) was assigned to each condition. The 1λ-1β-1δ model fit produced an adjusted *R*^2^ for the averaged data of 0.585, ranging from 0.292 to 0.782 across all participants. We next fit a 2λ-1β-1δ model to the data, in which one asymptote was assigned to the No Interpolation condition, and a second was assigned to the conditions with material intervening between the subject and the verb. This model fitting produced an adjusted *R*^2^ for the averaged data of 0.903, ranging from 0.823 to 0.945 across all participants. All participants showed an increase in adjusted *R*^2^ compared with the null model (average adjusted *R*^2^ increase = 0.337; minimum = 0.164; maximum = 0.641). The third fitting assigned a unique asymptote parameter to each Construction condition, a 3λ-1β-1δ model; this produced an adjusted *R*^2^ for the averaged data of 0.980, ranging from 0.955 to 0.993. The addition of an asymptote parameter again showed an increase in adjusted *R*^2^: compared to the 2λ-1β-1δ model, the average adjusted *R*^2^ increase was 0.074 (minimum = 0.029; maximum = 0.153); further, the average adjusted *R*^2^ increase was 0.411 (minimum = 0.192; maximum = 0.700) when this model was compared to the 1λ-1β-1δ model. The λ estimates (in *d*′ units) for the averaged 3λ-1β-1δ model were 4.06 for the No Interpolation condition, 2.58 for the Interpolated Object Relative condition, and 1.43 for the Interpolated Object + Subject Relative condition. An LMER analysis of the λ estimates showed a significant effect of Construction, *F*_(2, 8)_ = 104.74, *p* < 0.001. Pairwise comparisons closely tracked the pattern of the analysis of the empirical *d*′ data above. Specifically, the λ estimates for the No Interpolation condition were higher than both the Interpolated Object Relative (*t* = −8.16) and Interpolated Object + Subject Relative conditions (*t* = −14.43), and the Interpolated Object Relative condition λ estimate was greater than the Interpolated Object + Subject Relative estimate (*t* = −6.27). This finding—that a model with three asymptote parameters better fits the data than do models with two or one asymptote—is consistent with our analysis of the empirical *d*′ data. Thus, subsequent analyses focus on models with three asymptotes.

We next evaluated the effect of Construction on processing speed. Unlike the original study (McElree et al., 2003), the data do not suggest that these analyses should exclude speed differences in either the intercept or the rate; hence, we tested for differences manifesting in the intercept (δ); rate (β); and in both parameters together. We began by fitting a 3λ-1β-2δ model to the data, with potential speed differences assigned to the intercept. As in the asymptote comparisons, one parameter was assigned to the No Interpolation condition, and the second was assigned to the conditions with intervening material. This model produced an adjusted *R*^2^ for the average data of 0.992, ranging from 0.983 to 0.994 for individuals. All participants showed an increase in the adjusted *R*^2^ for the 3λ-1β-2δ over the 3λ-1β-1δ model (average increase = 0.014; minimum = 0.001, maximum = 0.028). We subsequently fit a 3λ-1β-3δ model to the data, but the addition of a third intercept parameter was not warranted: no participants showed an improved adjusted *R*^2^ for this model relative to the 3λ-1β-2δ model (adjusted *R*^2^ = 0.992; average increase = −0.002, minimum = −0.003, maximum = −0.001). Parameter estimates for the 3λ-1β-2δ are shown in Table 2.

**Table 2:** **Experiment 1: adjusted ***R***^2^, ***d***′s, and parameter estimates for the average data and individual participants for the 3λ-1β-2δ exponential model**.

	**Adj**.	***d*****-primes**			**Asymptotes**			**Rate**	**Intercept**	
	***R*^2^**	***d*′ 1**	***d*′ 2**	***d*′ 3**	**λ1**	**λ2**	**λ3**	**β1**	**δ1**	**δ2**
**Avg**	**0.992**	**3.58**	**2.55**	**1.45**	**3.897**	**2.690**	**1.489**	**0.826**	**0.831**	**1.223**
S1	0.983	3.83	2.55	1.18	3.975	2.416	1.038	1.358	0.733	1.306
S2	0.994	2.80	1.08	0.46	2.866	1.126	0.425	1.316	1.158	1.026
S3	0.989	3.28	2.58	1.83	4.129	3.271	2.359	0.519	0.948	1.433
S4	0.993	3.62	2.63	1.89	3.988	2.994	2.093	0.775	1.113	1.580
S5	0.985	4.35	3.90	1.89	4.776	3.943	1.947	0.891	0.728	0.973

We next evaluated potential speed differences that could result from the rate parameter, and fit a 3λ-2β-1δ model to the data. This model produced an adjusted *R*^2^ of 0.996 for the average data, ranging from 0.990 to 0.993 for individuals; however, data from two participants could not be fit to this model without overestimating the asymptote parameters. For those participants that were successfully fit with this model, the addition of a second rate parameter yielded an improved model fit over the 3λ-1β-1δ model (average adjusted *R*^2^ increase = 0.018; minimum = 0.001; maximum = 0.038). In addition, the 3λ-2β-1δ model fit the data better than the 3λ-1β-2δ model for two of these participants (average adjusted *R*^2^ increase = 0.008; minimum = 0.005; maximum = 0.010). A subsequent fit using a 3λ-3β-1δ model, excluding both participants without 3λ-2β-1δ model parameter estimates, showed that a third rate parameter was not warranted by the data (adjusted *R*^2^ = 0.996; average adjusted *R*^2^ increase = 0).

Finally, we considered a 3λ-2β-2δ model, in which speed differences could arise from both rate and intercept; one participant's data could not be modeled, again due to overestimated asymptotes, and was not included. Although this model (adjusted *R*^2^ = 0.997) did result in improved adjusted *R*^2^ for the average data relative to the model with two intercept parameters (3λ-1β-2δ; average adjusted *R*^2^ increase = 0.003; minimum = −0.001; maximum = 0.010), there was no adjusted *R*^2^ difference between this model and the 3λ-2β-1δ model (average adjusted *R*^2^ increase = 0; minimum = 0; maximum = 0.002).

LMER analyses of the parameter estimates for the models with two (3λ-2β-1δ, 3λ-2β-2δ; all *t*s < 1.7) and three [3λ-3β-1δ; *F*_(2, 6)_ = 2.41, *p* = 0.171] rate parameters were non-significant, possibly due to the small number of participants. However, the LMER analyses of the model with three intercept parameters (3λ-1β-3δ) revealed a significant main effect of Construction, *F*_(2, 8)_ = 6.31, *p* = 0.023). *T*-tests indicated that the No Interpolation condition was significantly different than both the Interpolated Object Relative condition (*t* = 2.84) and the Interpolated Object + Subject Relative condition (*t* = 3.27), but the two long conditions were not statistically different (*t* < 1). In addition, the LMER test of the 3λ-1β-2δ model confirmed that the two intercept parameters differed significantly (*t* = 2.576). These analyses are all consistent with the modeling conclusions that adding a third rate or intercept parameter is not warranted. Overall, these analyses indicate that the best model for both individual and average data is the 3λ-1β-2δ model: all participants were fit by this model, and alternative models did not yield consistent improvement. However, because our conclusions do not depend on whether the second speed parameter manifests on either the rate or the intercept, we present the parameter values for both models (see Tables 2, 3). The key conclusion is that there is no evidence to support the inclusion of a third speed parameter (either rate or intercept) for any participant or for the average data.

**Table 3:** **Experiment 1: adjusted ***R***^2^, ***d***′s, and parameter estimates for the average data and individual participants for the 3λ-2β-1δ exponential model**.

	**Adj**.	***d*****-primes**			**Asymptotes**			**Rate**		**Intercept**
	***R*^2^**	***d*′ 1**	***d*′ 2**	***d*′ 3**	**λ1**	**λ2**	**λ3**	**β1**	**β2**	**δ1**
**Avg**	**0.996**	**3.58**	**2.55**	**1.45**	**3.666**	**3.256**	**1.685**	**1.156**	**0.439**	**0.886**
S1	0.993	3.83	2.55	1.18	3.820	3.276	1.417	2.328	0.442	0.907
S2	0.993	2.80	1.08	0.46	2.879	1.097	0.412	1.270	1.752	1.144
S5	0.990	4.35	3.90	1.89	4.490	4.427	2.185	1.306	0.603	0.874

### Discussion

Consistent with previous research (e.g., McElree, 2000; McElree et al., 2003), we observed a negative correlation between response accuracy and the amount of material interpolated between the sentences' matrix verbs and the subject nouns. The significant differences in the empirical *d*′ data and in the model asymptotes confirm that as the distance between the subject and verb increases, the probability of accurately resolving the long-distance dependency decreases. Such asymptotic decreases are attributable to either an overall decrease in the quality of the memory representation over time, or to a decrease in the diagnostic distinctiveness of the retrieval cue (i.e., the featural characteristics of the verb) relative to the to-be-retrieved information (see Van Dyke and Johns, 2012). In addition, SAT response functions were best fit by a model in which there were two speed parameters, one reflecting fast access when no retrieval was necessary (i.e., the condition in which no material intervened between a verb and its grammatical head noun, leaving the most recently processed item active), and a second reflecting slower access when the critical item was not in focal attention and required retrieval. Critically, there was no benefit to including a third speed parameter (either on the rate or intercept), which would have supported a search-based retrieval mechanism: verbs retrieved their subjects with the same speed regardless of interpolated material. This pattern of asymptotic and dynamic differences is the characteristic signature of direct-access retrieval, and is apparent in the individual participants' data (see Table 2)^4^.

In addition, our participants' performance on conditions with grammatical anomalies in an embedded clause (conditions T5 and T8) suggests that they were not simply focusing on the initial noun and final verb in order to make their grammaticality judgments. Averaged correct rejection rates for these conditions for each of the response lags were 49.8, 50.4, 51.3, 54.9, 61.7, 64.3, 70.8, 75.0, 76.3, 78.4, 80.3, 82.3, 83.2, and 84.3%. Correct rejection rates for the corresponding experimental conditions (T4 and T7), in which the ungrammaticality derived from the sentence-final verb, were 49.6, 49.9, 50.7, 54.7, 63.1, 69.2, 75.6, 80.3, 81.9, 83.6, 84.1, 85.6, 85.2, 84.7%. As in McElree and colleagues' original study (McElree et al., 2003), accuracy was higher in the experimental conditions than in the conditions designed to discourage strategic processing. However, unlike the original study, correct rejection rates were not asymptotic at early lags in conditions T5 and T8; rather, the pattern of correct rejections seems to reflect an exponential response function. This difference could arise from any of the ways our study differs from the original, including our use of the multiple-response variant of the SAT technique, our use of auditory presentation of the sentences, or some combination of the two. For example: perhaps the relatively faster presentation of the sentences in an auditory (relative to the previously used visual) modality prevented early decision making. Alternatively, perhaps the need to process (at each response tone) an acceptable verb in light of an earlier anomaly, reduced participants' confidence in rejecting the sentence and/or prolonged repair routines aimed at finding a correct interpretation. However, such explanations are speculative, and ultimately are unrelated to the main issues addressed here. The value of these correct rejection rates is their clear demonstration that our participants processed the interpolated material, rather than simply ignoring it.

The results of this experiment replicate McElree and colleagues' demonstration of direct-access retrieval (McElree et al., 2003). These results are significant for three reasons. First, our MR-SAT replication of the original SR-SAT study continues a tradition of validating important findings about the operation of the human memory system across SAT techniques (e.g., McElree and Dosher's SR-SAT replication of Wickelgren and colleagues' MR-SAT findings regarding the focus of attention; Wickelgren et al., 1980; McElree and Dosher, 1989). Second, these results constitute the first evidence that, as in reading comprehension, collegiate comprehenders employ a content-addressable, direct-access retrieval mechanism during listening comprehension. Finally, unlike previous research, this interpretation is not susceptible to any confound related to orthographic processing. Thus, these results suggest that direct-access retrieval is a modality independent cognitive operation. Additionally, they validate the auditory MR-SAT procedure as an appropriate tool for investigating the retrieval mechanism in individual participants regardless of reading skill.

## Experiment 2

The results of our first experiment, in tandem with previous studies, suggest that direct-access retrieval may be the “default” setting during language comprehension, as it has now been observed both during reading and listening comprehension. Experiment 2 assessed the potential for direct-access retrieval in poor readers. Motivation for this work comes from studies indicating that capacity-based explanations are unlikely to account for poor reading comprehension (e.g., Traxler et al., 2012; Van Dyke et al., 2014; for review see Van Dyke and Johns, 2012). Rather, they point to limited capacity parsing architectures that rely on a fast, direct-access retrieval mechanism to restore information into the focus of attention as needed (e.g., Lewis and Vasishth, 2005; Lewis et al., 2006). However, studies establishing the presence of direct-access retrieval during language comprehension have been conducted exclusively with university students, presumably possessing a relatively high degree of comprehension skill. As such, this evidence only suggests that memory capacity is not important for argument integration in adult *skilled readers*. This leaves open the question of whether less-skilled readers are able to employ the same direct-access retrieval mechanism as skilled readers; that is, poor comprehension in these readers may arise because they simply do not have access to a direct-access retrieval mechanism, and must instead rely upon a slower, less efficient mode of retrieval (i.e., search) during comprehension. Numerous findings showing that less-skilled readers are typically slower than skilled readers to retrieve phonologically encoded information during comprehension support this possibility (Perfetti, 1985; Swan and Goswami, 1997a,b; Wolf and Bowers, 1999; Goswami, 2011).

Thus, our goal in Experiment 2 was to use the auditory SAT technique to determine whether less-skilled readers have access to an efficient, direct-access retrieval mechanism *at all*. The question of whether less-skilled readers are able to use direct-access retrieval is particularly important given that the prevailing account of memory limitations during reading comprehension suggests that poor readers' comprehension is inherently compromised—that reading skill is essentially pre-determined by fundamental, fixed differences in the memory system. The most obvious example of this approach is the notion of intrinsic, fixed WM capacities, which are thought to determine the facility with which a given comprehender may process linguistic information (e.g., Just and Carpenter, 1992; Caplan and Waters, 1999). According to this account, those with low WM capacities are predestined to be poor comprehenders, while those with higher WM capacities are not.

An alternative possibility is suggested by Ericsson and Kintsch (1995) in their Long-Term WM model. According to this model, skilled performance on any task (e.g., mental calculations, medical diagnosis, playing chess) is predicated on the development of highly efficient, skill-specific access structures, in which retrieval cues in active memory facilitate access to information in LTM. In each case, skilled practitioners enjoy rapid access to critical information, while those less-skilled will retrieve information more slowly and with difficulty. In the context of skilled reading comprehension, the development of proficient decoding, by which readers use orthographic representations to access lexical information, may provide the critical link between active and LTM. That is, because skilled readers have highly efficient mappings between the orthographic, phonological, and semantic characteristics of a word, they may enjoy direct-access retrieval of the lexical information upon which higher-level language processes (syntactic parsing, semantic, and discourse integration) depend. Less-skilled readers, in contrast, may instead be forced to rely on less efficient, search-based retrieval.

Critically, both of these accounts suggest that poor readers simply do not have access to an efficient retrieval mechanism to support reading—either because they do not (and cannot) have one, or because they do not have sufficient expertise to develop one. Thus, the importance of this experiment derives from its assessment of less-skilled readers' memory operations *when they are not reading*. If poor readers show the ability to employ content-addressable direct-access during auditory language processing, then they are not inherently saddled with a less efficient default retrieval mechanism. Furthermore, if less-skilled readers demonstrate the ability to use a direct-access retrieval mechanism, then it also cannot be the case that efficient retrieval is a byproduct of the development of reading expertise.

We used the same materials as in our first experiment. In addition, the participants in this study were not university students; we recruited a community-based sample of non-college bound young persons. Our previous experience with this population led us to expect large skill differences on a range of cognitive measures (e.g., Braze et al., 2007, in press; Shankweiler et al., 2008; Kuperman and Van Dyke, 2011; Magnuson et al., 2011; Johns et al., 2014; Van Dyke et al., 2014; Kukona et al., submitted). Our sample was age-matched to the standard college subject-pool population, which permits comparisons with previous studies of memory operations during language processing. As in those studies, we expected our participants' accuracy to vary according to the length of our experimental sentences (see Materials, Experiment 1), with the lowest accuracy in the longest conditions. As in Experiment 1, the critical comparisons for assessing the retrieval mechanism derive from the processing speed dynamics (rate and intercept) of their response functions. If poor readers use a search-based mechanism, then retrieval speed should vary as a function of the length of the experimental sentences (i.e., as a function of the amount of material interpolated between the matrix verb and its head noun). However, if poor readers are able to use a direct-access retrieval mechanism, speed should be fast when no retrieval is required (i.e., when there is no intervening material) and invariant across all other conditions, which do require retrieval.

### Method

#### Participants

Informed consent was obtained from 22 young people (ages 16–24) recruited from the local New Haven community. We recruited participants in a number of ways, including presentations at adult education centers, advertisements in local newspapers, flyers placed on adult school campuses, community centers, public transportation hubs, local retail and laundry facilities, and referrals from current and past study participants. All participants were right-handed native English speakers without a diagnosed reading or learning disability, and were paid for their participation ($20/h). Each participated in two 3-h experimental sessions identical to those described in Experiment 1, including initial training and an intersession period in which they completed additional cognitive assessments (for another study) and rested.

**Figure 2:**
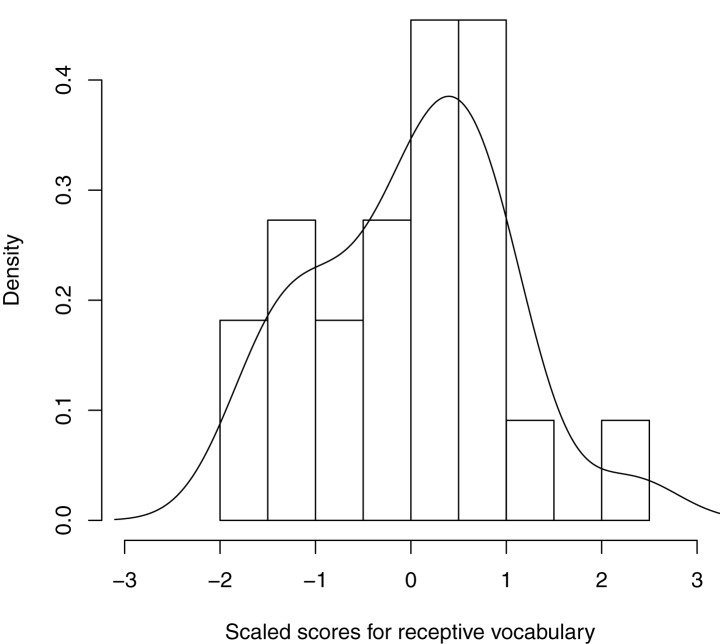
**Density histogram and curve of scaled scores for receptive vocabulary (Peabody Picture Vocabulary Test, 3E; Dunn and Dunn, 1997)**.

We assessed Reading Ability via the Peabody Picture Vocabulary Test (PPVT, 3E; Dunn and Dunn, 1997), which is a measure of receptive (i.e., interpretive, rather than productive) vocabulary. Vocabulary is known to be a limiting factor in the development of reading comprehension (Joshi, 2005; Perin, 2013). It frequently emerges as a unique predictor of reading ability, accounting for variance beyond that captured by other measures such as decoding, or by indices of reading comprehension (e.g., Braze et al., 2007, in press; Fraser and Conti-Ramsden, 2008; Ouellette and Beers, 2010; Tunmer and Chapman, 2012). There are now many psycholinguistic studies in which vocabulary was the critical measure for investigating individual differences in linguistic performance (e.g., Traxler and Tooley, 2007; Prat and Just, 2011; see also Long et al., 2008; Hamilton et al., 2013), including work from our lab using the PPVT (Braze et al., 2007, in press; Van Dyke et al., 2014). The distribution of scaled PPVT scores is shown in Figure 2; descriptive statistics and age equivalents are shown in Table 4. (Our participants completed the vocabulary assessment together with other skill assessments as part of a different study. We present a summary of these assessments in Table 4 so as to further characterize the cognitive abilities of this sample; however only the vocabulary assessment is used in the current analyses.)

**Table 4:** **Range, means, and standard deviations for selected cognitive battery measures**.

**Measure**		**Range**	***M***	***SD***	**Max. possible**
1	Receptive vocabulary	74–128	96.41	13.60	204
	*Age equivalent score*	10–22	16.67	4.66	22
2	Word reading (word attack)	18–32	25.73	3.45	32
	*Grade equivalent*	3.5–19	8.74	4.33	19
3	Word identification	54–74	65.18	5.70	76
	*Grade equivalent*	5.1–18	11.38	4.68	19
4	Reading fluency	56–94	75.45	11.86	98
	*Grade equivalent*	7.7–19	13.19	3.86	19
5	Reading comprehension	30–42	35.77	3.35	47
	*Grade equivalent*	4.3–19	10.52	4.73	19
6	Oral comprehension	19–30	25.59	2.77	34
	*Grade equivalent*	4.4–19	11.71	3.51	19
7	Gates-MacGinitie	27–46	36.82	5.58	48
	*Grade equivalent*	>PHS^*^	PHS		
8	Working memory capacity	24–57	42.55	8.38	60
9	IQ	63–123	94.18	13.87	–

* *Post High School*.

*Measure 1, Peabody Picture Vocabulary Test-Revised (Dunn and Dunn, 1997); 2–6, Woodcock-Johnson-III Tests of Achievement (Woodcock et al., 2001); 7, Gates-MacGinitie Reading Tests (MacGinitie et al., 2000); 8, listening span (Daneman and Carpenter, 1980); 9, Weschler Abbreviated Scales of Intelligence (Psychological Corporation, 1999)*.

#### Materials, procedure, data analysis

The materials, procedure, and parameters of the data analysis were identical to Experiment 1, except that the analyses included fixed effects of Reading Ability and the interaction of Reading Ability × Construction.

### Results

**Figure 3:**
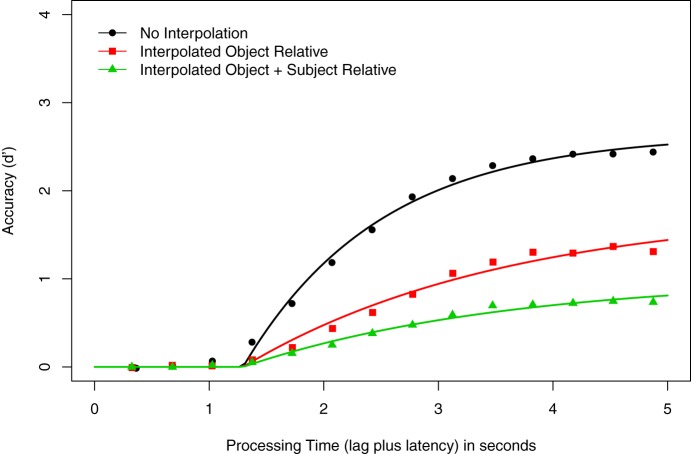
**Speed-accuracy tradeoff results for Experiment 2**. Average *d*′ accuracy as a function of processing time (in seconds) for the grammaticality judgments of sentences with the following constructions: no embedded material (circles), one object-relative clause (squares), and one object- and one subject-relative clause (triangles). Smooth curves show the best fitting 3λ-1β-2δ exponential model.

**Figure 4:**
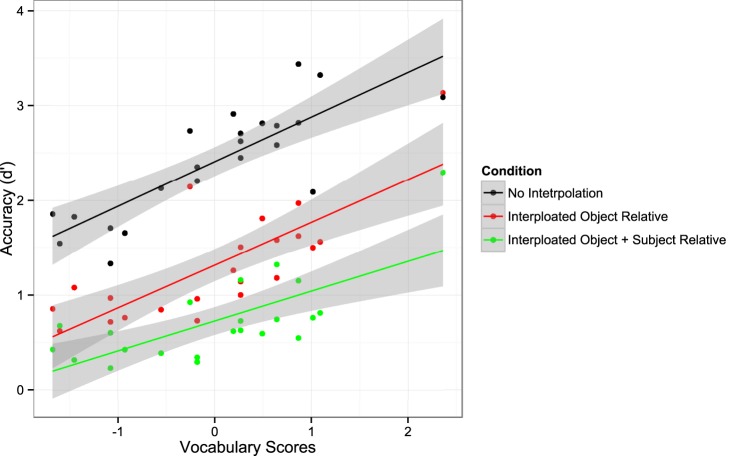
**The main effect of Reading Ability on ***d***′ accuracy in Experiment 2**.

Figure 3 shows the averaged *d*′ data (data points) and the best fitting 3λ-1β-2δ model (smoothed curves) as a function of processing time for the experimental conditions (No Interpolated Material, Interpolated Object Relative, Interpolated Object + Subject Relative). The LMER analysis of the mean of the last four *d*′ values yielded significant main effects of Construction, *F*_(2, 40)_ = 161.00, *p* < 0.001, and Reading Ability, *F*_(1, 20)_ = 56.11, *p* < 0.001. This effect is depicted in Figure 4. However, the interaction of Construction × Reading Ability was not significant, *F*_(2, 40)_ = 1.563, *p* = 0.222. Pairwise comparisons to resolve the main effect of Construction showed that accuracy was higher when there was no material between subject and verb (*d*′ = 2.41) than when there was an intervening object relative clause (*d*′ = 1.32), *t* = −11.57, or when there were intervening subject and object relative clauses (*d*′ = 0.73), *t* = −17.66. In addition, the asymptotic accuracy of the Interpolated Object Relative condition was significantly higher than that of the Interpolated Object + Subject Relative condition, *t* = −6.09. This pattern replicates the empirical *d*′ findings from both our first experiment and McElree et al. (2003).

Hierarchical modeling of the data proceeded as in the previous experiment, first comparing the 1λ-1β-1δ (null), 2λ-1β-1δ, and 3λ-1β-1δ models. The 1λ-1β-1δ model fit produced an adjusted *R*^2^ for the averaged data of 0.540, ranging from 0.299 to 0.895 across all participants. The 2λ-1β-1δ model (in which the additional asymptote parameter was again assigned to the conditions with interpolated material) produced an adjusted *R*^2^ for the averaged data of 0.947, ranging from 0.863 to 0.984 across all participants. All participants showed an increase in adjusted *R*^2^ compared with the null model (average adjusted *R*^2^ increase = 0.409; minimum = 0.03; maximum = 0.665). Finally, the 3λ-1β-1δ model produced an adjusted *R*^2^ for the averaged data of 0.990, ranging from 0.960 to 0.991 for individuals. Compared to the 1λ-1β-1δ model, the average adjusted *R*^2^ increase was 0.455 (minimum = 0.08; maximum = 0.692); compared to the 2λ-1β-1δ model, the average adjusted *R*^2^ increase was 0.046 (minimum = 0; maximum = 0.11). The λ estimates (in *d*′ units) for the averaged 3λ-1β-1δ model were 2.80 for the No Interpolation condition, 1.43 for the Interpolated Object Relative condition, and 0.80 for the Interpolated Object + Subject Relative condition. The LMER analysis of the λ estimates revealed significant main effects of Construction, *F*_(2, 40)_ = 196.66, *p* < 0.001, and Reading Ability, *F*_(1, 40)_ = 50.53, *p* < 0.001, but the interaction was again non-significant, *F*_(2, 40)_ = 2.24, *p* = 0.12. Pairwise comparisons to resolve the significant Construction effect closely tracked the pattern of the analysis of the empirical *d*′ data above. Specifically, the λ estimates for the No Interpolation condition were higher than both the Interpolated Object Relative (*t* = −13.34) and Interpolated Object + Subject Relative conditions (*t* = −19.38), and the Interpolated Object Relative condition λ estimate was greater than the Interpolated Object + Subject Relative condition (*t* = −6.04). This finding—that a model with three asymptote parameters better fits the data than do models with two or one asymptote, and that Reading Ability does not interact with this pattern—is consistent with our analysis of the empirical *d*′ data. Thus, our subsequent analyses again focused on models with three asymptotes.

We next evaluated the potential effects of Construction and Reading Ability on processing speed. It was first necessary to determine the best-fitting model for the average and individual data, so that each participant's rate (β) and intercept (δ) parameters could be examined in light of their scores on our vocabulary assessment. As in our first experiment, the data do not suggest that either the intercept or rate parameters can be excluded from analysis (see Figure 3). We first assigned an additional parameter to the intercept, so that the 3λ-1β-2δ model assigned one δ for the No Interpolation condition, and another for the conditions with intervening material. The adjusted *R*^2^ for this model's averaged data was 0.995, ranging from 0.969 to 0.994 for individuals. All participants but four showed an increase in the adjusted *R*^2^ for the 3λ-1β-2δ over the 3λ-1β-1δ model (average increase = 0.006; minimum = −0.001, maximum = 0.02). A subsequent fitting of a 3λ-1β-3δ model to the data showed that the addition of a third intercept parameter was not warranted: although eight participants showed an improved adjusted *R*^2^ for this model relative to the 3λ-1β-2δ model, on average the adjusted *R*^2^s were identical (adjusted *R*^2^=0.995; average increase = 0.001; minimum = −0.001, maximum = 0.007).

Next, we evaluated the rate parameter, adding a β so that one parameter was assigned to the No Interpolation condition, and the other to the conditions with interpolated material. This 3λ-2β-1δ model (average adjusted *R*^2^ = 0.995, individual adjusted *R*^2^ = 967 to 0.994) improved model fit over the 3λ-1β-1δ model: all but three participants showed an increase in adjusted *R*^2^ (average adjusted *R*^2^ increase = 0.006; minimum = 0; maximum = 0.034). However, this model was only a minimal improvement over the 3λ-1β-2δ model: although eight participants showed an increased adjusted *R*^2^ (average increase = 0.001; minimum = −0.004; maximum = 0.017), the remaining 14 showed either no improvement or a decrement in fit (from −0.001 to −0.004). Moreover, the adjusted *R*^2^ for the 3λ-2β-1δ model's average data was identical to the 3λ-1β-2δ model. A subsequent 3λ-3β-1δ model fitting indicated that a third rate parameter was not warranted by the data (adjusted *R*^2^ = 0.994; average adjusted *R*^2^ increase = 0.001, minimum = −0.001; maximum = 0.005). In light of this, the absence of a clear difference between the 3λ-2β-1δ and 3λ-1β-2δ models suggests that differences in retrieval speed may derive from the addition of either a second δ or β parameter, determined individually for each participant.

Finally, we considered a 3λ-2β-2δ model, in which speed differences could arise from both rate and intercept. This model (adjusted *R*^2^ = 0.995) was a slight improvement for nine participants (and a decrement for one participant) relative to the 3λ-1β-2δ model (average adjusted *R*^2^ increase = 0.001; minimum = −0.001; maximum = 0.016); it was also a slight improvement over the 3λ-2β-1δ model for eight participants (average adjusted *R*^2^ increase = 0.001; minimum = 0; maximum = 0.004). Of those participants showing an increased adjusted *R*^2^ with a 3λ-2β-2δ model, only two showed an increase relative to both of the models with six parameters.

Overall, this pattern of model fits makes two critical points. First, models with three parameters for either the rate or intercept are not appropriate for this data. Second, although it is clear that a model with two speed parameters is appropriate for this data, the additional parameter may manifest on the rate, the intercept, or potentially both indices of retrieval dynamics.

We conducted a series of LMER analyses of the β and δ estimates for the five models considered above. In addition, in order to determine whether our participants' retrieval dynamics varied according to reading skill, Reading Ability was included as a factor (and interaction term) in all comparisons where appropriate. However, across all models, there were no main effects or interactions associated with Reading Ability (3λ-3β-1δ, 3λ-1β-3δ: both *F*s < 1.5, lowest *p*-value = 0.158; 3λ-1β-2δ, 3λ-2β-1δ, 3λ-2β-2δ: all *t*s < 1.4). Therefore, all subsequent analyses focus only on the Construction factor.

The LMER analysis of the estimates for the model with three rate parameters (3λ-3β-1δ) was non-significant, *F*_(2, 40)_ = 1.39, *p* = 0.259. For the model with three intercept parameters (3λ-1β-3δ), the LMER test revealed a main effect of Construction, *F*_(2, 40)_ = 14.21, *p* < 0.001. Subsequent *t*-tests revealed that the intercept parameters for the No Interpolation condition were significantly different than both the Interpolated Object Relative (*t* = 4.72) and Interpolated Object + Subject Relative conditions (*t* = 4.51); but the intercepts in the two long conditions (Interpolated Object Relative and Interpolated Object + Subject Relative) were not significantly different (*t* < 1). Both LMER analyses indicate that the addition of a third dynamics parameter is not warranted, and that only models with two dynamics parameters are justified.

We now turn to the models with two dynamics parameter estimates. The more conservative of these models only have six parameters (i.e., 3λ parameters, and 3 parameters divided between the β and δ). *T*-tests confirm a significant difference between the intercepts in the 3λ-1β-2δ model (*t* = 6.41) and between the rates in the 3λ-2β-1δ model (*t* = −2.76). For the 3λ-2β-2δ model, *t*-test revealed that the difference between the rate parameter estimates was non-significant (*t* = −1.14); however, the intercept estimates differed significantly (*t* = 3.62). Thus, a conservative interpretation of the current pattern of results suggests that the 3λ-1β-2δ model should be preferred (see Table 5 for both the average and the individual parameter estimates for this model).

**Table 5:** **Experiment 1: adjusted ***R***^2^, ***d***′s, and parameter estimates for the average data and individual participants for the 3λ-1β-2δ exponential model**.

	**Adj**.	***d*****-primes**			**Asymptotes**			**Rate**	**Intercept**	
	***R*^2^**	***d*′ 1**	***d*′ 2**	***d*′ 3**	**λ1**	**λ2**	**λ3**	**β1**	**δ1**	**δ2**
**Avg**	**0.995**	**2.41**	**1.32**	**0.73**	**2.690**	**1.482**	**0.834**	**0.779**	**1.266**	**1.579**
S1	0.978	2.79	1.58	1.32	3.114	1.817	1.534	0.684	1.195	1.287
S2	0.977	3.44	1.97	1.15	3.534	1.759	1.110	1.572	0.895	1.550
S3	0.980	2.20	0.96	0.35	2.363	1.040	0.440	1.058	1.429	1.990
S4	0.980	1.55	0.62	0.68	1.640	0.641	0.677	3.608	0.876	1.214
S5	0.963	1.34	0.72	0.23	1.402	0.729	0.400	3.386	1.563	1.951
S6	0.984	2.71	1.14	0.73	3.097	1.337	0.882	0.771	1.547	1.893
S7	0.979	2.73	2.15	0.93	2.836	2.128	1.010	1.719	1.827	2.282
S8	0.992	1.71	0.97	0.60	2.007	1.203	0.755	0.972	2.258	2.583
S9	0.986	2.09	1.50	0.76	2.057	1.554	0.708	4.848	2.957	2.814
S10	0.984	1.83	1.08	0.32	1.915	1.172	0.400	1.074	1.353	1.938
S11	0.982	3.09	3.13	2.29	3.287	3.276	2.388	0.964	0.982	1.248
S12	0.969	2.59	1.18	0.75	2.776	1.382	0.875	1.223	2.148	2.490
S13	0.993	2.35	0.73	0.30	2.315	0.757	0.400	2.367	1.335	1.602
S14	0.982	3.32	1.56	0.81	3.365	1.524	0.720	2.252	1.717	1.714
S15	0.984	2.45	1.00	1.16	2.812	1.214	1.283	0.772	1.626	1.687
S16	0.991	2.82	1.62	0.55	2.938	1.560	0.567	3.810	1.601	1.684
S17	0.984	1.86	0.86	0.43	1.930	0.890	0.481	1.751	2.250	2.291
S18	0.982	2.13	0.85	0.39	2.126	0.818	0.400	1.690	1.177	1.292
S19	0.988	1.66	0.76	0.43	1.699	0.759	0.482	1.405	1.594	1.882
S20	0.987	2.91	1.26	0.62	3.179	1.375	0.652	1.065	1.601	1.848
S21	0.989	2.81	1.81	0.60	3.104	2.003	0.710	1.126	2.026	2.383
S22	0.988	2.63	1.51	0.63	2.898	1.765	0.668	0.876	1.466	1.747

### Discussion

The results of this experiment replicate both Experiment 1 and the SR-SAT experiment in McElree et al. (2003). Analyses of both the *d*′ and model asymptote estimates confirm that response accuracy decreased linearly in relation to the amount of material that intervened between sentential NPs and matrix verbs. Thus, as in previous studies, processing the additional interpolated material decreases the likelihood of retrieving the correct constituent and/or mis-parsing the syntactic relations among sentence constituents. These possibilities arise because the additional material either negatively affects the representation of the target constituent, or else the additional material (i.e., the introduction of additional NPs) decreases the diagnostic value of the matrix verbs' retrieval cues (McElree et al., 2003; see also Van Dyke and McElree, 2011; Van Dyke and Johns, 2012). In addition, we also observed individual differences in both *d*′ and asymptotic accuracy based on Reading Ability, such that higher ability was associated with more accurate overall performance. However, there was no interaction of Reading Ability with the amount of interpolated material. Thus, the interpretation of the effect of Reading Ability is straightforward: more skilled readers were able to more accurately resolve the subject-verb dependency than less skilled readers, regardless of distance between subject and verb.

We also observed speed dynamics differences showing that access to the critical item was fastest when there was no interpolated material between noun and verb (i.e., when no retrieval was necessary); and, when intervening material necessitated retrieval, the speed of access did not vary according to how much material intervened between noun and verb. Both the modeling and the inferential statistics indicate that retrieval speed is invariant, regardless of the amount of embedded material. In addition, although we observed differences related to Reading Ability in accuracy measures, we observed no effect (or interaction) of Reading Ability with any index of retrieval dynamics. That is, readers retrieved information that was outside focal attention with equal speed, regardless of Reading Ability.

As in Experiment 1, there is important independent evidence that participants were processing the embedded material. Correct rejection rate at each response lag for the conditions with the anomaly within the interpolated region (T5 and T8) was 49.9, 49.7, 49.6, 51.0, 52.4, 53.5, 54.5, 56.4, 55.8, 56.4, 61.2, 61.2, 61.7, and 60.4%. Correct rejection rates for the corresponding conditions containing a sentence-final ungrammaticality were 49.9, 50.2, 50.3, 51.4, 53.3, 56.9, 60.3, 64.0, 66.5, 68.4, 69.6, 69.8, 71.5, and 71.7%. This pattern is identical to that observed in Experiment 1: overall accuracy is higher in the experimental conditions, and responses to the control conditions appearing to follow an exponential function. One distinction between the two experiments is that these rejection rates—although still clearly above chance for all conditions—are lower than those in the first experiment. This is consistent with the overall performance of the participants in this experiment, who had considerably lower *d*'s in every condition than the university students in Experiment 1, and is undoubtedly a function of the broader range of reading ability.

This pattern of results—in which accuracy differs systematically according to the amount of material interpolated between the retrieval cue and the to-be-accessed item, but retrieval speed does not—is once again consistent only with content-addressable, direct-access retrieval. Thus, this experiment provides the first evidence that memory capacity is not important for argument integration in both skilled and less-skilled readers during listening comprehension. In addition, based on these results, the slowing associated with poor reading comprehension (e.g., Perfetti, 1985; Swan and Goswami, 1997a,b; Wolf and Bowers, 1999; Goswami, 2011) cannot be directly attributed to the absence of an efficient mechanism for retrieving critical information from memory. That is, the direct-access retrieval mechanism that is thought to subserve basic memory operations (see Clark and Gronlund, 1996), and which has been observed during language processing in collegiate readers (e.g., McElree et al., 2003; for review see McElree, 2015) was not innately compromised in our sample of less-skilled readers. These results also indicate that direct-access retrieval is not the result of increasingly proficient reading ability, as many of our participants had low word reading and comprehension ability (see Table 4). This suggests that a model of retrieval from LTM based on task-specific expertise (e.g., Ericsson and Kintsch, 1995) does not support argument integration during routine language processing. Rather, the pattern of results we observed suggests that individual variation in language processing is driven by the quality of the representation to be retrieved, and not the mechanism by which it is retrieved (Van Dyke and Shankweiler, 2013). This conclusion is bolstered by the use of the auditory SAT procedure: none of our effects can be attributed to either felicitous or impaired processing based on orthographic information (Harm and Seidenberg, 2004).

## General discussion

These experiments contribute to the growing body of evidence in support of cue-based direct-access retrieval as the memory mechanism supporting argument integration during online sentence processing. Both of our experiments demonstrate the signature pattern of direct-access retrieval: variation in accuracy based on dependency distance, but constant retrieval speed when a distal constituent is required to complete a long-distance dependency. Our results replicate previous findings that suggest that a direct-access retrieval mechanism supports online parsing operations (McElree et al., 2003; see also McElree, 2000; Martin and McElree, 2008, 2009, 2011; Van Dyke and McElree, 2011). Our results also extend previous findings, as we are the first to report that this type of mechanism supports comprehension of spoken language. As such, these studies suggest that direct-access retrieval is modality independent. Consequently, they further suggest that this retrieval mechanism, long known to subserve basic memory operations outside the domain of linguistic processing, may also be a core property of the human language faculty (see also McElree, 2015).

Our findings with respect to reading ability are consistent with this possibility. The results of Experiment 2 confirm that poor readers do not *de novo* employ a qualitatively different memory mechanism than that used by good comprehenders. Moreover, the use of the SAT methodology allows us to make several nuanced (and, perhaps, surprising) claims with respect to poor reading ability. For example, that we observed no main effects or interactions of Reading Ability on indices of retrieval speed may be unexpected in light of the many previous reports of lower fluency and slower reading rates in poor readers (for reviews see Torgesen et al., 2001; Chard et al., 2002); models of reading frequently attribute such behavior to impaired speed of retrieval (e.g., LaBerge and Samuels, 1974; Stanovich, 2000). However, because standard fluency measures capture both speed and the overall quality of readers' interaction with a text (Adams, 1990; Ashby et al., 2013), they do not take into consideration the speed-accuracy tradeoffs inherent in any timed assessment. Accordingly, it is not possible to clearly distinguish the contributions of representation quality and memory access speed to reading speed measures with traditional assessments.

In contrast, the implication of our results are clear: differences in representational quality, rather than in retrieval speed, contribute more to a comprehender's performance. Specifically, all our effects of Reading Ability were found only on the asymptote, which is understood within the SAT literature as an index of representation quality (e.g., memory strength; see Dosher, 1979; Wickelgren et al., 1980). Indeed, readers are known to differ in their ability to differentiate memory representations along various dimensions, with skilled readers able to make fine-grained distinctions that less skilled readers cannot (Perfetti and Hart, 2002; Perfetti et al., 2005; Landi and Perfetti, 2007; Perfetti, 2007; see also Long and Prat, 2008). Clinical reports showing that dyslexic readers are less able to make linguistically relevant phonetic distinctions compared to age-matched reading-level controls (e.g., Bogliotti et al., 2008; Goswami et al., 2011) are also consistent with this interpretation. Finally, there is also evidence that interventions that specifically attempt to increase reading speed are largely unsuccessful (Torgesen et al., 2001; Berends and Reitsma, 2005; Marinus et al., 2012), unless the intervention seeks to strengthen the representation of specific words or word parts (Mattingly, 1972; National Reading Panel, 2000; Thaler et al., 2004; Conrad and Levy, 2011). Findings such as these support the argument that representational quality is the crucial determinant of whether a given representation will be available for argument integration (e.g., Perfetti and Hart, 2002; Perfetti, 2007; Perfetti et al., 2008; Frishkoff et al., 2011).

Our observation of direct-access retrieval in our poor readers has important implications for the study of, and remediation of, reading difficulty and disability. Although the current study of auditory sentence processing does not demonstrate that poor readers employ direct access during reading, it does demonstrate that direct-access retrieval is not inherently “broken” or unavailable to these readers. This suggests that, like skilled readers, they are eligible to use a parsing architecture characterized by a severely limited active memory and an efficient direct access retrieval mechanism (Lewis et al., 2006). Because all readers, regardless of skill, have the minimal capacity required by such a system—the most recently processed item—inherent differences in WM capacity cannot be the source of comprehension difficulty, at least with respect to basic argument integration. Further support for this position comes from our recent study of a community-based sample of adult readers, in which comprehension of visual sentences was related not to WM capacity but, as in our second experiment, to receptive vocabulary (Van Dyke et al., 2014). Other recent work, in which first-grade children's development of reading comprehension skill was tracked before, during, and after intensive training on WM tasks, is similarly consistent: even when WM performance increased significantly, there was no measurable effect on the children's development of reading comprehension skill (Fuchs et al., 2014; see also Banales et al., 2015).

Poor quality lexical representations have a particularly serious impact on the efficiency of direct-access retrieval, wherein retrieval cues must be able to uniquely identify target representations. If representations do not instantiate important or relevant distinctions, then the mapping between cue and target will be indeterminate, leading to retrieval of incorrect representations. This situation has been studied extensively in the memory domain under the rubric of “cue-overload” (e.g., Watkins and Watkins, 1975) and has also been referred to as retrieval interference (see Van Dyke and Johns, 2012 for a review). Van Dyke and McElree (2006) demonstrated this effect in the language domain using a dual task paradigm (see also Gordon et al., 2002). Participants read sentences such as these:

(8a) It was the boat that the guy who lived by the sea *sailed* over two sunny days.
(8b) It was the boat that the guy who lived by the sea *fixed* over two sunny days.

For each sentence, a memory load was either present or absent; if present, participants received a short list of words to memorize prior to reading the sentence (e.g., TABLE-SINK-TRUCK). The presence of retrieval interference was determined by the main verb. In conditions such as (8a), the verb *sailed* is not overloaded: because the memory list words are not “sail-able,” the verb's semantic cues are able to uniquely identify the displaced subject NP *boat*. However, in conditions such as (8b), the verb *fixed* is an overloaded retrieval cue: that is, because the semantic cues provided by *fixed* are not uniquely diagnostic of its target in memory, the “fixable” items in the memory list compete with the “fixable” target in the sentence. Van Dyke and McElree found, in university students, that cue-overload increased reading difficulty at the verb—an effect which disappeared when the competing matches in the memory list were absent. (Similar effects in reading paradigms without a dual task have also been reported; e.g., Gordon et al., 2001; Van Dyke and Lewis, 2003; Van Dyke, 2007.) A subsequent study, using the same paradigm and materials with a community-based sample of participants, found that readers' sensitivity to interference induced by overloaded retrieval cues varied negatively with receptive vocabulary (indexed, as in our SAT experiment, by PPVT; Van Dyke et al., 2014). In that study, low vocabulary scores were uniquely predictive of greater interference effects, including online reading difficulty and impaired performance on offline comprehension questions. Van Dyke and colleagues proposed that such readers—many of whom also had low scores on a range of other linguistic skill measures—were likely to have lexical representations in which important distinctions (on orthographic, phonologic, and/or semantic dimensions) were absent. It is precisely these distinctions that could be crucial for discriminating among similar, competing lexical representations when a retrieval cue is overloaded.

Van Dyke et al. (2014) were the first to report that poor readers were more vulnerable to retrieval interference than skilled readers. However, the association between low verbal ability and effects related to the strength or quality of representations, rather than retrieval speed, is also broadly consistent with a recent SAT study examining individual differences in interference resolution in recognition memory (Öztekin and McElree, 2010). Using an extreme groups design, Öztekin and McElree assessed recognition of words that were either present in a studied list; absent from, but consistent with the semantic categories of, studied list items (“distant negatives”); or absent from the studied list, but nonetheless present in the immediately preceding study list (“recent negatives”). As in the current study, there were no individual differences associated with retrieval speed, which was invariant for all items but the most recently processed list word. Also as reported here, individual differences emerged only on the SAT parameter associated with representation quality: low ability participants had lower asymptotic accuracy. This difference was driven by low ability participants' greater rate of false alarms to the recent negative lure trials. Öztekin and McElree suggested that this greater susceptibility to interference could result from lower-quality representations, or from the impaired ability to distinguish between information based on familiarity and episodic details (i.e., cue-overload). As this study also used the SAT method, we take these results as important corroborating evidence for our own position: namely, that individual differences have their effect on measures of representation quality (or strength), and not on retrieval speed^5^. Taken together, these studies converge on the notion that it is the probability of retrieving the necessary item, determined by qualitative properties of the item's representation, that is a crucial determinant of reading ability—rather than intrinsic capacity differences, or the absence of an efficient retrieval mechanism.

Finally, as this is the first time the SAT method has been used to examine individual differences in language processing, we acknowledge that the suggestion that poor reading ability may be unrelated to slowed retrieval should be treated cautiously. Moreover, although the size of the current sample is in line with other published SAT studies, it would be desirable to replicate our study with an even larger sample to verify our results with respect to speed parameters. However, it is important to note that the main conclusion from this study is actually entirely orthogonal to whether poor reading ability is associated with slower retrieval speed. The crucial finding here is that regardless of reading ability, retrieval speed was unaffected by the amount of interpolated material between the target subject and its verb. The fact that the speed to access the target subject in our longest condition (Interpolated Object + Subject Relative Clause condition) was the same as that for accessing the target in the shorter Interpolated Object Relative Clause condition means that these retrievals occurred without executing a backwards sequential search through the contents of memory. Rather, all participants employed a direct-access retrieval mechanism irrespective of Reading Ability. Thus, even if we had observed a main effect of ability on speed parameters, this would have only attested to the possibility that retrieval was slower overall. This would have said nothing about the presence or absence of a direct-access retrieval mechanism in poor comprehenders.

The experiments reported here validate the auditory SAT procedure as a useful, highly sensitive tool for investigating the architecture of language comprehension across individuals with widely varying linguistic abilities. Because it gauges performance in the auditory modality, the procedure is not susceptible to problems related to inefficient orthographic decoding skills that confound other online assessments. This opens up new possibilities for investigations of memory access during language processing to special populations, such as adolescents with poor reading comprehension, dyslexics, spoken language bilinguals (e.g., heritage language speakers), or functionally illiterate language users. In addition, because longer, multi-sentence and passage-length materials have been difficult to implement in the visual SAT paradigm, our findings suggest the possibility of investigating memory retrieval during the online processing of discourse-level dependencies. Especially considered alongside the potential to investigate the influence of a broader range cognitive abilities on the dynamics and accuracy of memory retrieval during online language comprehension, the results of this study raise many exciting possibilities for future research.

### Conflict of interest statement

The authors declare that the research was conducted in the absence of any commercial or financial relationships that could be construed as a potential conflict of interest.

## Footnotes

^1^There are two known circumstances, both task-specific, in which multiple items may be in focal attention: the task must either promote the “chunking” of information (McElree, 1998), or encourage participants to actively maintain distant information (McElree, 2001, 2006). The connection between these findings and language operations has not been systematically explored. The only attempt of which we are aware is unpublished data suggesting that some phrase types (PP, adverbial) do not displace information in focal attention (Wagers and McElree, 2009; discussed in McElree, 2015). However, as these structures are not examined in our experiments, we adopt the formulation that is most consistent with findings from published language research: that a single word—the most recently processed item—is maintained in focal attention.

^2^McElree et al. (2003) also included a condition containing two embedded object-relative clauses, of comparable length to items such as (7), which also contain two embedded clauses (a subject-relative and an object-relative). The double-object condition yielded both significantly lower response accuracy and a significantly slower rate of retrieval than all other conditions. McElree and colleagues discussed these findings at length, noting that the data pattern remains inconsistent with a search-based explanation; we refer interested readers to McElree et al. (pp. 81–82).

^3^Available from GitHub (https://github.com/). Contact matsukk@mcmaster.ca for details.

^4^One participant, S2, shows speed dynamics contrary to the predicted direction. This is also true of one participant in Experiment 2 (S9). We believe this to be an artifact of the fitting function: modeling the dynamic portion of the SAT function can be difficult if *d*′ scores are very low (McElree, personal communication). However, because these participants' asymptotic accuracy is consistent with both the other participants and the pattern observed in the original study (McElree et al., 2003), and out of consideration for the sample sizes in both experiments, we ultimately elected to include these data in our analyses. If these participants are excluded, our results do not change—and indeed become stronger in the predicted direction.

^5^Öztekin and McElree (2010) used a working memory assessment as their only skill measure. There is much evidence for high correlations between this assessment and other language and reading-related measures, including vocabulary, phonological processing, decoding ability, rapid naming, reading fluency, and spoken language ability. Hence, we prefer to interpret this result as referring to a more general verbal skill ability rather than about working memory capacity per se. See Van Dyke et al. (2014) for further discussion of this issue.
